# No evidence for phenotypic condition-dependent ejaculate allocation in response to sperm competition in a seed beetle

**DOI:** 10.1093/beheco/arae095

**Published:** 2024-11-20

**Authors:** Blake W Wyber, Joseph L Tomkins, Leigh W Simmons

**Affiliations:** Centre for Evolutionary Biology, School of Biological Sciences, The University of Western Australia, 35 Stirling Highway, Perth 6009, Australia; Centre for Evolutionary Biology, School of Biological Sciences, The University of Western Australia, 35 Stirling Highway, Perth 6009, Australia; Centre for Evolutionary Biology, School of Biological Sciences, The University of Western Australia, 35 Stirling Highway, Perth 6009, Australia

**Keywords:** *Callosobruchus maculatus*, condition dependence, phenotypic plasticity, sperm competition risk, strategic ejaculation

## Abstract

Sperm competition is known to favor the evolution of male traits that confer an advantage in gaining fertilizations when females mate multiply. Ejaculate production can be costly and the strategic allocation of sperm in relation to the sperm competition environment is a taxonomically widespread phenomenon. However, variation among males in their ability to adjust ejaculate allocation has rarely been explored. Here, we manipulated the phenotypic condition of male seed beetles, *Callosobruchus maculatus*, via larval diet quality and measured ejaculate allocation across varying levels of sperm competition manipulated using olfactory cues. Furthermore, we asked how strategic ejaculation was impacted by previous ejaculation. We found no variation in ejaculate allocation in response to experimentally manipulated cues to sperm competition. Ejaculate allocation was reduced by a male’s previous mating history but was unaffected by the larval diets on which males were reared. We suggest that either male seed beetles are unable to adjust ejaculate size to the immediate competitive environment, or that sperm displacement strategies employed by males favor maximal investment at all mating events, especially when unmated females are infrequently encountered. As our study is one of few to examine condition dependence in strategic ejaculation, emphasis should be placed on future studies investigating this possibility across a wider range of taxa and animal mating systems.

## Teaser

Ejaculates are expensive to produce and males are well known for their capacity to adjust the size of their ejaculates in response to the competitive environment. Recent research suggests that the ability of males to respond to rivals may depend on available ejaculate resources. We tested this idea in a seed beetle. Although males became depleted of ejaculate after mating, resource availability did not affect ejaculate allocation in response to rival cues.

## Introduction

Polyandry is widespread across sexually reproducing taxa (Arnqvist and Nilsson 2000; Simmons 2005; Pizzari and Wedell 2013). Sperm competition theory predicts that selection should favor any trait in males that confers an advantage in competition for fertilization (Parker 1970; Simmons 2001), and sperm competition has been shown to be a significant factor shaping male expenditure on ejaculate production and allocation (Parker and Pizzari 2010; Simmons and Fitzpatrick 2012; Lüpold et al. 2020).

Given that ejaculate production is costly (Macartney et al. 2019), theory predicts that males should adjust their expenditure on, and allocation of, ejaculates in response to the risk and intensity of sperm competition (Gage 1991; Engqvist and Reinhold 2006; Parker and Pizzari 2010; Kelly and Jennions 2011; Wilson et al. 2014). When males perceive a risk of sperm competition (the probability that females will mate with at least one other male), they are predicted to increase their ejaculate allocation to compete with rival sperm (Parker and Pizzari 2010). Conversely, males are predicted to allocate less ejaculate as sperm competition intensity increases (the number of males competing for fertilizations) because the marginal fitness gain from ejaculate allocation should decline with increasing numbers of competing males (Engqvist and Reinhold 2006; Parker and Pizzari 2010). Empirical studies have provided considerable support for these predictions, finding that males adjust the overall quantity of ejaculate (Gage 1991; Gage and Baker 1991), sperm quality (Simmons et al. 2007; Thomas and Simmons 2007), sperm quantity (Cook and Gage 1995), seminal fluid composition (Ramm et al. 2015; Bartlett et al. 2017), and sperm morphology (Immler et al. 2010; Iwata et al. 2021), in response to cues in the sociosexual environment indicative of competition.

The impact of phenotypic condition on ejaculate production is now well documented (Rahman et al. 2013; Macartney et al. 2018; Simmons et al. 2022). Numerous studies that manipulate phenotypic condition via dietary limitation have found marked reductions in ejaculate traits, including ejaculate mass (Watanabe and Hirota 1999; Ferkau and Fischer 2006; Perry and Rowe 2010), sperm number and morphology (Rahman et al. 2013; Kahrl and Cox 2015; Kaldun and Otti 2016), and sperm viability (Evans et al. 2015; Ng et al. 2018). However, whether nutrient availability affects how individuals partition ejaculate resources among mating events has only recently been considered (Wylde et al. 2019; Lymbery et al. 2022). It has been suggested that only males in high phenotypic condition have the resources necessary to facultatively raise ejaculate allocation in response to competition, while those in low condition, with limited resources, maybe unable to respond accordingly. There are just 3 studies that have addressed the phenotypic condition dependence of strategic ejaculation. When the phenotypic condition of male neriid flies (*Telostylinus angusticollis*) was manipulated via larval diet quality, focal males in high, but not low phenotypic condition provided larger ejaculates to singly-mated females than to unmated females (Wylde et al. 2019). Thus, for neriid flies, male responses to sperm competition risk do appear to depend on phenotypic condition. However, for guppies (*Poecilia reticulata*), neither diet quality (Lymbery et al. 2022) nor rearing temperature (Chung et al. 2024) effect patterns of strategic ejaculation, although genetic condition (inbreeding) does (Chung et al. 2024). Here, we build on these studies by asking if plasticity in ejaculate allocation depends on phenotypic condition in seed beetles, *Callosobruchus maculatus*.

Seed beetles have become a widely utilized model system for sexual selection research (Zuk et al. 2014). Males of this species are notable for the large size of their ejaculates, particularly their first ejaculate which can account for 8% to 10% of total body mass (Fox et al. 1995a; Hotzy and Arnqvist 2009). The size of ejaculates in this species exceed that required to fertilize available ova (Eady 1995; Savalli and Fox 1999; Edvardsson and Tregenza 2005). There is evidence to suggest that larger ejaculates serve to secure greater paternity share by displacing the sperm of rival males (Eady 1991, 1995). Large ejaculates may also act to reduce female remating receptivity (Savalli and Fox 1999). However, when males engage in successive copulations they suffer dramatic reductions in sperm quantity and total ejaculate weight (Eady 1995; Savalli and Fox 1999; Wilson et al. 2014). Thus, prior mating leaves males ejaculate depleted, with fewer reserves available for subsequent matings. Male seed beetles have been shown to plastically adjust their copulatory behavior (Wilson and Tomkins 2014; Wilson et al. 2014) and decrease ejaculate allocation in response to the presence of rival males (Lymbery et al. 2019). In general, male insects have been found to be capable of gauging the sociosexual context of particular mating events, specifically the risk and intensity of sperm competition, using cues that include cuticular hydrocarbons (CHCs) transferred to females by their previous mates (Thomas and Simmons 2008; Bretman et al. 2011; Lane et al. 2015; House et al. 2024), and male seed beetles have been shown to plastically moderate their harmfulness to females during mating when exposed to the CHC profiles of familiar and unfamiliar rival males (Lymbery et al. 2019).

We examined plasticity in ejaculate allocation by male seed beetles that varied in their phenotypic condition and immediate sperm reserves. We altered the phenotypic condition of focal males through the manipulation of larval diet quality and modified the perceived competitive environment through CHC perfuming. We examined male responses to social cues on their first and second matings, to identify how sperm depletion might impact strategic ejaculation. We used 2 estimates of ejaculate allocation. First, we estimated the weight of ejaculates transferred to females at mating. Second, given that ejaculate size has known effects on female productivity in seed beetles (Fox et al. 1995b; Savalli and Fox 1999; Moya-Larano and Fox 2006), we estimated female productivity following mating as a complimentary measure of male allocation. We predicted that rival male CHCs would induce plasticity in ejaculate allocation. Specifically, risk models of sperm competition predict that the presence of CHCs from one other male should increase ejaculate allocation, while intensity models of sperm competition predict that increasing numbers of rival male CHC profiles should reduce ejaculate allocation (Parker and Pizzari 2010). If strategic ejaculation is dependent on phenotypic condition, we predicted that males raised on a high-quality larval diet would show greater plasticity in ejaculate allocation in response to olfactory cues of sperm competition compared with males raised on a low-quality larval diet, and that males mating for a second time should exhibit less plasticity due to the prior depletion of ejaculate reserves.

## Methods

### Study population

The source population of *C. maculatus* used in this study was derived from populations collected by the CSIRO in 2005. Stock populations were held in a climate-controlled room set at 26 °C with a 12:12-h light/dark cycle and provided with mung beans (*Vigna radiata*) as a breeding substrate.

### Larval diets

Female seed beetles deposit their eggs on the surface of dried legumes, within which larvae feed and mature into adults. Adults are facultatively aphagous, so that nutrient uptake can occur exclusively in the larval stage of development (Małek et al. 2019). Thus, the life history of this beetle is well suited for the manipulation of adult condition via larval nutrition. Condition was manipulated by providing either a high- or low-quality laying substrate to female *C. maculatus* during oviposition. The substrates used were mung beans (*Vigna radiate*) and chickpeas (*Cicer arietinum*). Individuals raised on chickpeas have been shown previously to have severely reduced fecundity and viability compared to individuals raised on mung bean (Price et al. 2017) so that chickpeas were considered a low-quality diet and mung beans a high-quality diet, respectively.

Larval diet treatments were established by isolating ~100 mung beans from the stock population that each had just one egg attached. Beans were housed individually within a ventilated 1.5 mL Eppendorf tube. Once adult beetles emerged, two 20:20 male:female groups were provided with 300 g of either chickpeas or mung beans. Approximately 2 wk after adult beetles were introduced to their respective laying substrates, beans with a single egg attached were collected. To ensure focal individuals remained unmated, beans were isolated in ventilated plastic tubes. Tubes were inspected daily for emerging individuals throughout the duration of the experiment. A total of 160 focal males were collected; 80 raised on mung beans, and 80 raised on chickpeas.

### CHC perfuming

**Fig. 1.**
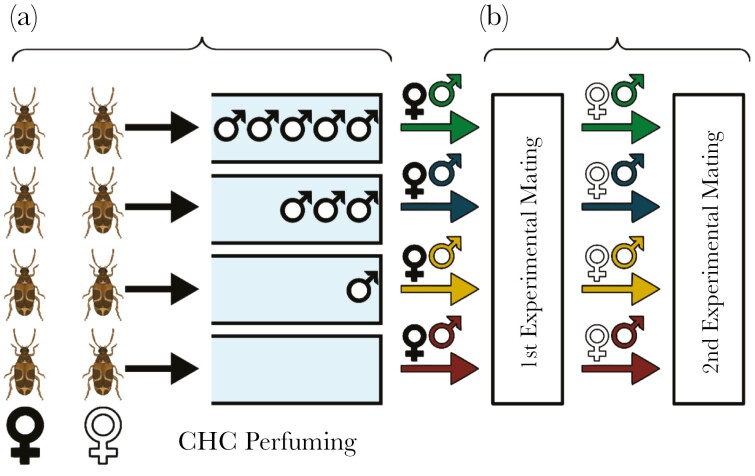
—Experimental design: (a) Pairs of females were assigned to a single vial, and were perfumed with the CHC extracts of 0, 1, 3, or 5 males. (b) A single focal male was sequentially mated to 2 females. Black symbols (♀) represent the first female mated to a focal male, while white symbols represent the second female.

Cuticular hydrocarbon “perfuming” was employed to experimentally manipulate male perception of both the risk and intensity of sperm competition. We included 4 CHC treatment levels, wherein females were either uncoated or coated with the CHC profiles of one, 3 or 5 unrelated “donor” males (Fig. 1a). Unmated males were collected from the stock populations for use as CHC donors and were euthanized by freezing. These individuals were isolated while still within beans, to ensure that no transfer of CHCs occurred between beetles.

Each donor male was thawed and immersed for 5 min in 0.2 mL of hexane in a glass vial, after which the male was removed and the hexane allowed to evaporate. CHCs were resuspended in 0.2 mL of hexane and vials were gathered into groups of 1, 3, or 5. In the single-male groups, the hexane was transferred into a fresh vial and allowed to evaporate. For the 3- and 5-male groups, the hexane from individual vials was combined into a single vial and mixed thoroughly. A 0.2-mL sample of the mixed CHCs was then pipetted into a fresh vial and the hexane evaporated. Thus, extracts were of the same CHC concentration but were derived from 1, 3, or 5 males. For the zero CHC treatment, 0.2 mL of hexane was left to evaporate within an empty glass vial. Once all hexane had evaporated, 2 unmated stock females were assigned to each of the CHC-free and CHC-coated vials (Fig. 1a). Each female was spun separately within the vial for 30 s using a Vortex. The female intended for the first mating was vortexed first, and the female assigned for the second mating was vortexed second (Fig. 1b).

### Experimental matings

Focal males were randomly assigned to one of the 4 CHC manipulation treatments (Fig. 1b). Males were provided with perfumed females 24 h post-emergence in sequential order (Fig. 1b). Males were weighed before introduction to the first female. Immediately after copulation, mated females were re-weighed again to obtain an estimate of transferred ejaculate weight. Weights were estimated using a high precision Sartorius SE2 Micro Balance, to a precision of 0.0001 mg. Females were introduced to a 100 mL plastic container with 25 g of mung beans and allowed to oviposite. Containers were monitored for the emergence of offspring, and 2 wk after the first offspring emerged containers were frozen to standardize time allowed for offspring emergence, and to prevent the hatching of second-generation offspring. Emerged offspring were counted and used as a measure of female productivity. A total of 20 individuals were assigned to each larval diet by CHC profile treatment combination.

Some estimates of ejaculate weight were negative (i.e. females were lighter post-copulation). Negative ejaculate weights could have represented measurement error. However, the Sartorius SE2 was highly repeatable in its measurement; 2 repeated measures of the same 10 unmated females returned a repeatability estimate of ~1. The mean [95% CI] absolute change in weight between 2 measures of the same unmated female was just 0.0013 mg [0.0017, 0.0008], which is 0.15% of the mean change in weight recorded after mating 0.0946 mg [0.1067, 0.0823]. Thus, we assumed that a negative value most likely reflected an ejaculate too small for measurement. However, in some cases, males may have failed to ejaculate because the eggs subsequently laid by the female were not fertilized. When negative ejaculate weights co-occurred with females producing no offspring, the males were removed from the analysis. A total of 5 males were removed for ejaculation failure with their first mate, and 12 for ejaculation failure with their second mate. An additional 54 males were lost during the experiment because they failed to mate with both of their females (32), or their female mating partners were lost or died before laying eggs (22), leaving a sample of 89 focal males for statistical analysis (distributed across the diet (high or low) and CHC (0, 1, 3, 5) treatments thus: high 12, 8, 12, 9; low 13, 11, 12, 12).

### Statistical analyses

All statistical analyses were conducted in R (R_Core_Team 2021). Shapiro-Wilk’s and Bartlett’s tests were used to test for normality and homogeneity of variances, respectively. Ejaculate size was non-normally distributed with homogenous variance, but female productivity was found to violate both assumptions. Data were inspected visually for outliers. When outliers were suspected, those outside 2 absolute deviations around the median were excluded from the analysis (Leys et al. 2013). A total of 3 outliers were identified in the ejaculate dataset, 2 males raised on mung beans and one on chickpeas, and these were removed from further analysis, leaving a final sample size of 86. Data could not be normalized via transformation, and thus randomization tests were employed to confirm the conclusions drawn from linear models were robust (see below).

Repeated-measures Gaussian linear mixed models were used for both ejaculate weight and female productivity, using the “nlme” package (Pinheiro et al. 2023). We included larval diet, CHC treatment and mating order as fixed effects, and the pre-mating weight of both the male and female as covariates. Male identification number was included as a random effect to account for the repeated measures design. Mating order was included as a random slope to allow for the relationship of the first and second ejaculate to vary between individuals. All interactions between fixed effects were included in the full model and were removed if nonsignificant. The interactions between CHC treatment and larval diet and between CHC treatment and mating order were kept in the final models because these interactions represent the test of our hypothesis that plasticity in ejaculate allocation is condition dependent:


$$\begin{array}{l} \begin{array}{lllllll} & Ejaculate∼Diet+CHC\;treatment\; & \;\;\;+Mating\;N^{o}+Fem.\;W.+Male\;\;W.+\;\left(SC\;*\;Diet\right)\;\;\; & \;\;+\left(SC*Mating\;N^{o}\right)+(Mating\;N^{o}\;|\;ID)\;\;\; & Of\;fspring∼Diet+CHC\;treatment+Mating\;N^{o}\; & \;+\;Fem.\;\;W.+Male\;W.+\;\left(SC*Diet\right)\; & \;+\left(SC*Mating\;\;N^{o}\right)+(Mating\;N^{o}\;|\;ID)\; \end{array} \end{array}$$

We performed randomization tests on these final models. Randomization tests were conducted by randomly re-assigning relevant trait values (i.e. ejaculate size, or number of offspring) across the explanatory variables and calculating chi-square values. A total of 10, 000 randomizations were conducted per model and a *P*-value was calculated as the proportion of times the randomized chi-square values were greater than the observed chi-square value.

## Results

**Fig. 2.**
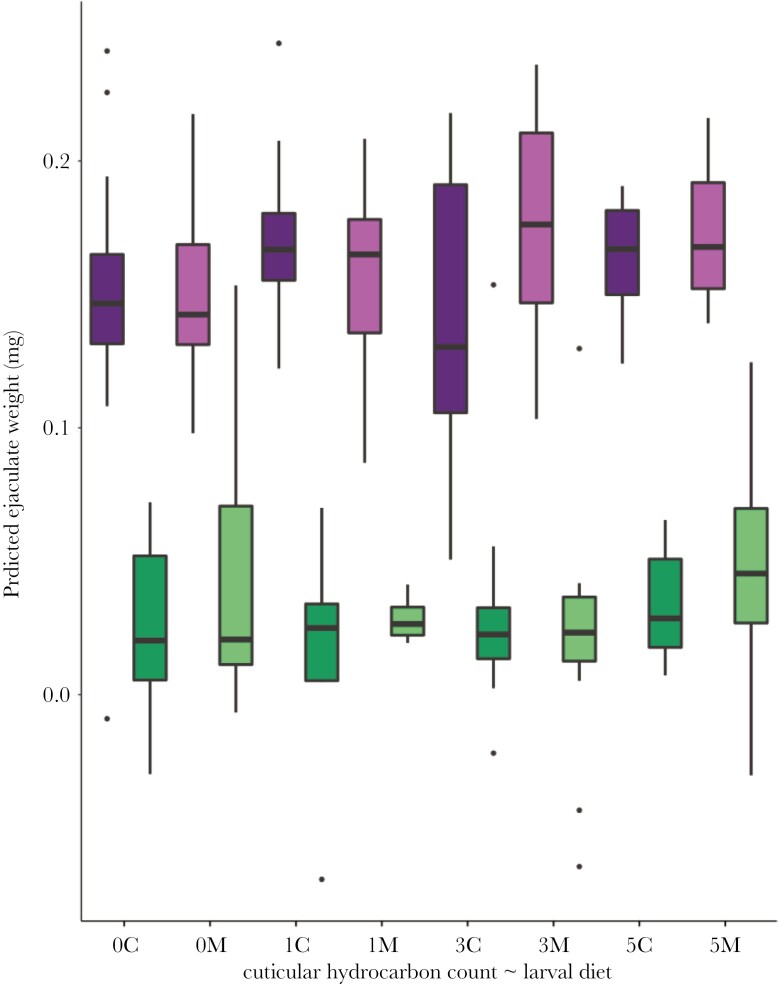
Box plots of the weight of ejaculate transferred when differing numbers of rival cuticular hydrocarbon profiles (0, 1, 3, and 5) were present on a male’s mating partner. Ejaculates from the first mating event (purple) and second mating (green) are shown, for males reared on a larval diet of chickpeas (dark, C) and mung beans (pale, M). Boxes represent the upper and lower quartiles, the line represents the median and the whiskers the upper and lower extremes excluding potential outliers which are indicated as closed circles.

Larval diet quality affected the pre-mating weight of males in the expected direction (chickpea: 3.11 ± 0.05 mg, mung beans:3.24 ± 0.05; *t* = 2.72, df 84, *P* = 0.059). Although larval diet affected male condition, the diet of focal males had no impact on the weight of ejaculate transferred to females (Table 1). Ejaculate allocated to females was unaffected by the number of rival CHC profiles present at the time of mating (Fig. 2). Mating order had a significant effect on ejaculate weight, where males transferred less ejaculate on their second mating (Table 1; Fig. 2). Neither the interaction between larval diet and CHC treatment, nor the interaction between mating order and CHC treatment, were found to be significant (Table 1).

**Table 1.** Repeated measures permutational analysis of variance for fixed effects and interactions on transferred ejaculate weight (*N* = 86, M-mung bean, C-chickpea).

	Estimate	CI (0.95)	χ^2^	df	*P*	*P* rand.
Larval diet (M-C)	0.005	−0.01, 0.02	1.69	1	0.19	0.22
CHC treatment	0.002	−0.00, 0.01	2.25	1	0.13	0.15
Mate order (2nd to 1st)	−0.13	−0.15, −0.11	364.67	1	<0.001^***^	<0.001^***^
Female Weight	−0.006	−0.01, 0.00	2.17	1	0.14	0.16
Male Weight	0.004	−0.01, 0.02	0.29	1	0.59	0.61
Diet × CHC treatment.	0.001	−0.00, 0.01	0.13	1	0.72	0.73
Mate Order × CHC treatment.	−0.001	−0.01, 0.01	0.12	1	0.72	0.73

**Fig. 3.**
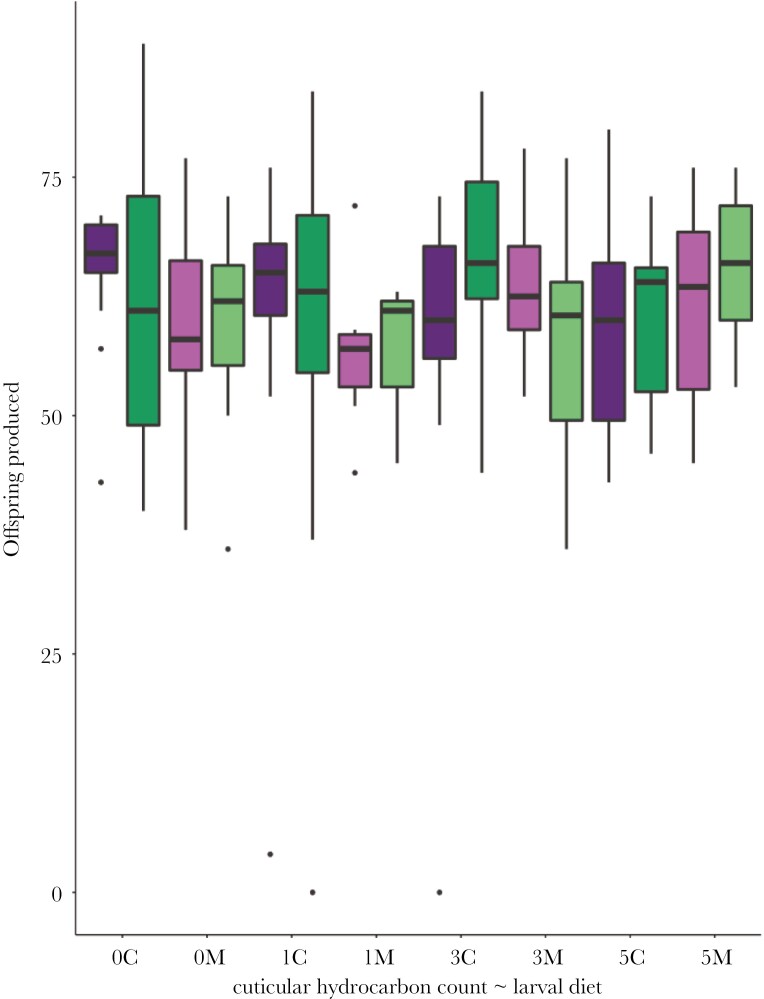
Box plots of the productivity of females when perfumed with differing numbers of rival cuticular hydrocarbon profiles (0, 1, 3, and 5). Productivities of first mating (purple) and second mating (green) females are shown, when the mating males were reared on a larval diet of chickpeas (dark, C) and mung beans (pale, M). Boxes represent the upper and lower quartiles, the line represents the median and the whiskers the upper and lower extremes excluding potential outliers which are indicated as closed circles.

The number of offspring produced by females was unaffected by male larval diet and CHC treatment (Table 2). Neither the mating order of females nor the weight of females had an impact on female productivity. The interaction between CHC treatment and larval diet was non-significant, as was the interaction between CHC treatment and mating order. No other significant interactions were detected (Fig. 3).

**Table 2.** —Repeated-measures permutational analysis of variance for fixed effects on female productivity (*N* = 86, M-mung bean, C-chickpea).

	Estimate	CI (0.95)	χ^2^	df	*P*	*P* rand.
Larval diet (M-C)	−2.18	−8.37, −4.00	0.02	1	0.88	0.89
CHC treatment.	−0.68	−2.40, −1.03	0.08	1	0.78	0.79
Mate order (2nd to 1st)	−0.03	−4.32, −4.25	1.06	1	0.30	0.31
Female Weight	8.87	6.43, 11.32	52.3	1	<0.001^***^	<0.001^***^
Male Weight	4.71	−1.87, 11.30	2.03	1	0.15	0.18
Diet × CHC treatment.	0.87	−1.27, 3.00	0.65	1	0.42	0.48
Mate Order × CHC treatment.	0.70	−0.79, 2.19	0.87	1	0.35	0.36

## Discussion

Although larval diet affected adult male size, we found no significant effect of larval diet quality on ejaculate size. Moreover, male seed beetles did not alter their ejaculate allocation patterns when exposed to cues signaling the risk and intensity of sperm competition. While ejaculate size was significantly depleted following a male’s first mating, ejaculate depletion did not influence how males allocated subsequent ejaculates when exposed to cues of sperm competition risk and intensity. Finally, we found no differences in female productivity as a result of male larval diet, ejaculate depletion, or our manipulations of sperm competition risk and intensity.

Male *C. maculatus* did not alter ejaculate allocation in the presence of rival male CHC profiles. Facultative alterations of ejaculate size in response to perceived sperm competition have been reported in previous studies of seed beetles drawn from the same populations that we used in the current study (van Lieshout et al. 2014; Lymbery et al. 2019). When males were exposed to either male- or female-biased social environments, those exposed to a greater proportion of rival males reduced their ejaculate allocation (van Lieshout et al. 2014). Although Lymbery et al. (2019) found that males exposed to rivals across multiple days reduced ejaculate allocation in relation to the accumulated number of rivals encountered previously, consistent with our study they did not find an effect of the immediate presence of rivals on ejaculate allocation. Similarly, although Wilson et al. (2014) reported longer mating durations when rival males were actually present, total ejaculate size transferred during mating remained unchanged. Collectively, these studies suggest that male seed beetles do not alter their current ejaculate size in response to the immediate social context. Given that focal individuals in our study were kept in complete pre-mating isolation, a male’s initial ejaculate may have been tailored for an environment reflecting no sperm competition, perhaps explaining the lack of response to the CHC treatments that we observed.

An alternative explanation is that our manipulation of cuticular hydrocarbons may have been insufficient to alert males to an increase in sperm competition risk or intensity. A previous study utilizing the fruit fly *Drosophila melanogaster* found that the detection of rivals by males, and thereby their strategic response, was dependent on the quantity of cues available for perception (Bretman et al. 2011). Male flies were systematically denied auditory, olfactory, tactile, and visual cues, followed by all possible cue removal combinations. When males are provided multiple cues, the efficacy of male responses to perceived sperm competition increased (Bretman et al. 2011). Males provided with the most cues were able to plastically adjust mating duration, and consequently fathered more offspring. However, manipulations of CHC olfactory cues alone were insufficient to provoke plastic responses to the presence of a rival in *D. melanogaster* (Bretman et al. 2011). Previous studies of *C. maculatus* have identified plasticity in mating behavior when males are able to directly interact with rivals, and thereby have access to all possible sensory cues (Wilson et al. 2014; Lymbery et al. 2019). Nevertheless, it has been found that seed beetle males mating in the presence of familiar and related CHC profiles reduce the harm to females during mating (Lymbery et al. 2019), suggesting that olfactory cues alone should have been sufficient to induce strategic ejaculation in our study.

The lack of plasticity in ejaculate size that we report may also be attributable to the mechanism of sperm competition in *C. maculatus*. Sperm competition risk and intensity models are based on the assumption that sperm competition conforms to a fair raffle, wherein each sperm has an equal chance of fertilization, and male fertilization success is proportional to the sperm invested by that male relative to the total sperm across all ejaculates received by a female (Parker and Pizzari 2010). For some systems, this assumption may not be valid. For example, for some insects the capacity of sperm storage organs can be limited such that males displace the sperm stored from rival males during mating (Parker and Pizzari 2010). Indeed, seed beetles employ indirect sperm displacement, whereby males displace the sperm of rivals through the transfer of large ejaculates, resulting in a strong last-male bias in paternity (Eady 1994). For sperm displacement systems, allocation strategies are better predicted from optimization models that are relatively independent of the number of sperm stored from rival males (Parker and Pizzari 2010). Consequently, ejaculate allocation should be relatively insensitive to the intensity of sperm competition in species with sperm displacement. Allocation nevertheless might be plastically altered on the basis of female mating status because minimal allocation is required to fill the sperm stores of a previously unmated female. As such, we might expect males to supply less ejaculate to virgin females compared to mated females (Parker et al. 1993; Parker and Pizzari 2010). We found that male seed beetles allocated ejaculates of equal weight to control females as to females perfumed with male CHCs, suggesting they may not discriminate between mated and unmated females. The same is true for yellow dung flies (*Scatophaga stercoraria*), where males displace rival sperm during copulation and copula duration is unaffected by female mating status, a situation that is likely due to the low frequency of encounters with unmated females in natural populations (Parker et al. 1993). Seed beetles typically live in high-density populations and suffer high levels of harassment from courting males (den Hollander and Gwynne 2009; Gay et al. 2009; Bacon and Barbosa 2020). Females may thereby be exposed to the CHCs of multiple males even when not mating with them so that male derived CHCs are unreliable as cues to a female’s mating history. For seed beetles, therefore, it may be that large population sizes and high mating frequencies (Ursprung et al. 2009) generate very weak selection for plasticity in ejaculate allocation (Parker et al. 1993).

Larval diet quality affected adult male size, but had no effect on ejaculate weight. This result was unexpected as meta-analysis has found that the production of ejaculate components is generally affected by resource abundance and quality (Macartney et al. 2019). Previous studies have investigated the condition dependence of sexually selected traits in *C. maculatus*, all of which have manipulated the phenotypic condition of individuals via larval diet quantity, wherein larvae were reared either in once-used or fresh mung beans (Cayetano and Bonduriansky 2015; Iglesias-Carrasco et al. 2018). Female fecundity was found to depend on the quantity of nutrients available during a female’s larval development, as well as the diet consumed by a female’s mating partner (Iglesias-Carrasco et al. 2018). Moreover, it was found that transferred ejaculate size was significantly smaller when males were reared on once-used beans compared to those reared on fresh beans (Iglesias-Carrasco et al. 2018). A notable difference between our study and previous studies is the method by which we attempted to manipulate male condition. Here, we manipulated the quality of diet, rather than quantity available for consumption. We used this method because the use of previously exploited beans would have exposed our subject males to social cues in the form of CHCs left within used beans. It is possible that the reduced ejaculate size of low-condition males found by Iglesias-Carrasco et al. (2018) could have been a plastic response to the perceived social environment, rather than evidence for condition dependence in ejaculate size. Given that we were interested in measuring strategic responses of males to their immediate mating environment we needed to avoid potential confounding effects arising from our manipulation of diet.

We found no relationship between male phenotypic condition and strategic ejaculation patterns. This result is consistent with that of Lymbery et al. (2022), who found that ejaculate allocation strategies of male Guppies (*P. reticulata*) were unaffected by food quantity manipulations. However, our findings conflict with the only other study to investigate the condition dependence of plastic ejaculate allocation in an insect (Wylde et al. 2019). Male neriid flies in high condition, but not low condition, responded to an increased risk of sperm competition by providing larger ejaculates to singly mated females compared to virgins (Wylde et al. 2019). Given that ejaculate traits are widely known to be impacted negatively by poor nutrition (Macartney et al. 2019), the results of Wylde et al. (2019) suggest that males in poor condition are resource-limited, and thus unable to increase their allocation of ejaculate resources in the face of competition. Ejaculate size in *C. maculatus* has been shown to decrease substantially across multiple mating events (Savalli and Fox 1999). Consistent with previous findings, our data show a marked reduction in ejaculate size following the first copulation. Nevertheless, sperm depletion did not affect male responses to sperm competition cues. We conclude therefore, that resource limitation, either in the form of reduced phenotypic condition, or due to ejaculate depletion, does not impact how male seed beetles partition their ejaculates among females.

Large females produced more offspring than their smaller counterparts, a pattern found previously in seed beetles, and insects generally (Wilson et al. 1999; Bonduriansky 2001). Even so, male *C. maculatus* did not appear to plastically adjust ejaculate allocation on the basis of female size. This result is consistent with a previous study which reported that male allocation patterns remained unaffected by the weight of females (Edvardsson and Tregenza 2005). Should males be able assess mate quality, and the variance in female quality is substantial, selection is expected to favor male choosiness, both overtly (Bonduriansky 2001), and cryptically through differential ejaculate allocation (Galvarni and Johnstone 1998; Reinhold et al. 2002). However, we found that males did not facultatively allocate their ejaculates based on female weight, suggesting that males of this species do not employ cryptic male choice.

In conclusion, we found no evidence to suggest that phenotypic condition altered the ejaculate allocation strategies of male seed beetles. Ejaculate depletion likewise had no impact on ejaculate allocation responses to sperm competition. It is now widely appreciated that males can and do tailor their ejaculates in response to the competitive environment. However, our study is only the third to explore phenotypic condition-dependence of this phenomenon. Although we found no evidence for condition dependent strategic ejaculation by seed beetles, future studies of a diversity of invertebrate and vertebrate taxa, with a diversity of sperm utilization mechanisms, will be required to fully evaluate the possibility that male plasticity in ejaculate allocation strategies can be influenced by the availability of ejaculate resources.

## Data Availability

Analyses reported in this article can be reproduced using the data provided by Wyber et al. (2024).
